# Human Coronavirus Photodynamic Inactivation and In Silico Mechanisms Induced by Ga(III) vs. Zn(II) Phthalocyanines

**DOI:** 10.3390/v18080815

**Published:** 2026-07-24

**Authors:** Neli Vilhelmova-Ilieva, Emilio Mateev, Muhammed Tilahun Muhammed, Aleksandra Rangelova, Diana Braikova, Ivan Iliev, Vanya Mantareva

**Affiliations:** 1 Department of Virology, Stephan Angeloff Institute of Microbiology, Bulgarian Academy of Sciences, 26 Georgi Bonchev, 1113 Sofia, Bulgaria; alexandraharalampieva@gmail.com; 2 Department of Pharmaceutical Chemistry, Faculty of Pharmacy, Medical University, 1000 Sofia, Bulgaria; e.mateev@pharmfac.mu-sofia.bg; 3 Department of Pharmaceutical Chemistry, Faculty of Pharmacy, Suleyman Demirel University, Isparta 32260, Turkey; muhammedmuhammed@sdu.edu.tr; 4 National Diagnostic and Research Veterinary Medical Institute, Bul. “Pencho Slaveikov” 15, 1606 Sofia, Bulgaria; 5 Institute of Organic Chemistry with Centre of Phytochemistry, Bulgarian Academy of Sciences, Bld. 9, 1113 Sofia, Bulgaria; diana.braykova@orgchm.bas.bg; 6 Institute of Experimental Morphology, Pathology and Anthropology with Museum, Bulgarian Academy of Sciences, 1113 Sofia, Bulgaria; taparsky@abv.bg

**Keywords:** HCoV-OC43, viral replication, virucidal effect, viral adsorption, enveloped viruses, phthalocyanines, photodynamic inactivation, molecular docking

## Abstract

Photodynamic inactivation (PDI) is a relatively new approach for targeting viruses. Recently, PDI has been shown to be effective against various viral infections, with a low probability of resistance development. The study presents PDI towards the infectivity of human coronavirus (HCoV-OC43) at different stages of its propagation and through different mechanisms of action. Two tetra-methylpyridiloxy-substituted gallium and zinc phthalocyanines (GaPcMe and ZnPcMe), exposed to light from a 660 nm light-emitting diode (LED), were evaluated and showed a high potential against HcoV-OC43. The effect of PDI on extracellular virions and the stage of their adsorption was assessed using the finite dilution method and by determining changes in viral infectivity (Δlgs). The impact on the viral replicative cycle was assessed by inhibition of the cytopathic effect (CPE). The direct effect on virions was notable for both phthalocyanines, but was significantly more pronounced for ZnPcMe (Δlg = 4). A strong inhibitory effect on virus adsorption was observed for ZnPcMe (from Δlg = 3.5 to complete inhibition, Δlg = 5.0, depending on the irradiation time). Both GaPcMe and ZnPcMe demonstrate PDI at an early stage of the virus replication cycle. This is reflected in the high photoinactivation indices PII = 44.0 for ZnPcMe and PII = 23.3 for GaPcMe. The binding potential of GaPcMe and ZnPcMe toward viral and host protein targets was investigated using molecular docking and molecular dynamics (MD) simulations. The computational panel included HCoV-OC43 3CLpro (Nsp5, PDB 9PAM) and homologous SARS-CoV-2 proteins, namely Nsp5, Nsp12, M protein, S protein, as well as human ACE2. The docking results indicated that both phthalocyanines exhibit notable theoretical binding affinity toward these targets.

## 1. Introduction

Photodynamic inactivation (PDI) of viruses has seen significant progress in recent years as a potential means of limiting the spread of the actual coronaviruses [1]. A general photosensitization mechanism begins with irradiation of a photosensitizer (PS) in an oxygen-containing environment. As a result, the generated singlet oxygen and other reactive oxygen species tend to damage biomolecules, including viral nucleic acids, lipids, and proteins. The effect is direct and depends mainly on the capacity of prooxidants to inactivate viruses, which cannot be repaired, as in eukaryotes and prokaryotes. PDI is used for periodontal therapy, root canal disinfection, denture stomatitis, fungal skin infections [2] and a number of viral skin infections caused by human papilloma virus (HPV), herpes simplex virus (HSV), varicella-zoster virus (VZV), and molluscum contagiosum virus (MCV) [3]. Well-known PSs, such as methylene blue (MB) and curcumin, induce PDI in various viral models [4,5,6]. Enhancing the activity of methylene blue as a PS has been achieved by a nanotherapeutic platform (MBSD) based on methylene blue nanoparticles stabilized with a primary fatty acid, a natural component of human nasal mucosa, for intranasal PDI. Illumination of MBSD significantly reduces viral gene expression and infectivity in multiple RNA and DNA viruses, including influenza A, SARS-CoV-2 variants (B.1 and Delta), Zika, Vaccinia, and emerging paramyxoviruses [7]. However, they require blue light for PS excitation and have limited tissue penetration, encouraging the development of a new generation of PSs, including phthalocyanines, for antiviral PDI applications. In a recent study, the cationic octakis(cholinyl)zinc phthalocyanine was evaluated for its effectiveness against SARS-CoV-2 isolates [8]. The phenothiazine compound MB was also reported to be effective against HCoV-OC43 and SARS-CoV-2 in an in vitro study, achieving 6-log inactivation [9]. An early study on coronaviruses evaluated a near-infrared-absorbing dye (DTTPB), showing targeting of the lipid bilayer in both viruses (HCoV-OC43 and HCoV-229E) [10]. This study reported a decrease in HCoV-OC43 protein expression in host cells. The results clearly demonstrated that DTTPB can effectively prevent HCoV-OC43 infection and its spread via PDI. A novel Ru(II) photo-dynamic complex (PDC), studied on influenza HA spike proteins, was cleaved off virion particles, as shown in the study by Coombs et al. [11]. Three isoquinolinium-based PSs (TPE-IQ, TPE-IQ-2O, and LIQ-TPE) were used in PDI on viruses (FMDV, HCoV-OC43, and HCoV-229E) with high efficiency, thereby reducing RNA copies in host cells [12]. This study was carried out following the protocol at a low power density (9 mW·cm^−2^), which suggested a practical application of PDI against coronavirus. Genetically correlated to the human coronavirus (HCoV-OC43), the bovine coronaviruses (BCoVs) were inactivated by cationic PSs such as Zn-PcChol8+ and MB [13]. A decrease in BCoV titers and in the morphological structure of viral particles was observed across various PDI protocols. The morphological changes included loss of spikes, changes in shape, a decrease in viral particle size, partial or complete destruction of the envelope, and complete disintegration of viruses. In the above study, a bovine coronavirus BCoV was evaluated as more sensitive to MB, which is the well-known approved PS-PDI. The mechanism is PS- and light-specific rather than target-dependent [14]. 

The present study aims to evaluate the effects of PDI with phthalocyanine complexes (ZnPcMe and GaPcMe) on human coronavirus (HCoV-OC43) in vitro under LED-660 nm exposure at different stages of viral reproduction. The virucidal effect on extracellular HCoV-OC43 virions, on the adsorption stage of viral particles to host cells (Vero E6), and on the intracellular replicative cycle of HCoV-OC43 was selected to assess PDI efficiency. The possible protein-level interaction mechanisms underlying the observed effects were explored using in silico modeling, including docking and molecular dynamics simulations on HCoV-OC43 3CLpro and selected SARS-CoV-2 and host protein targets.

## 2. Materials and Methods

### 2.1. Phthalocyanines and Other Chemicals

**Scheme 1:**
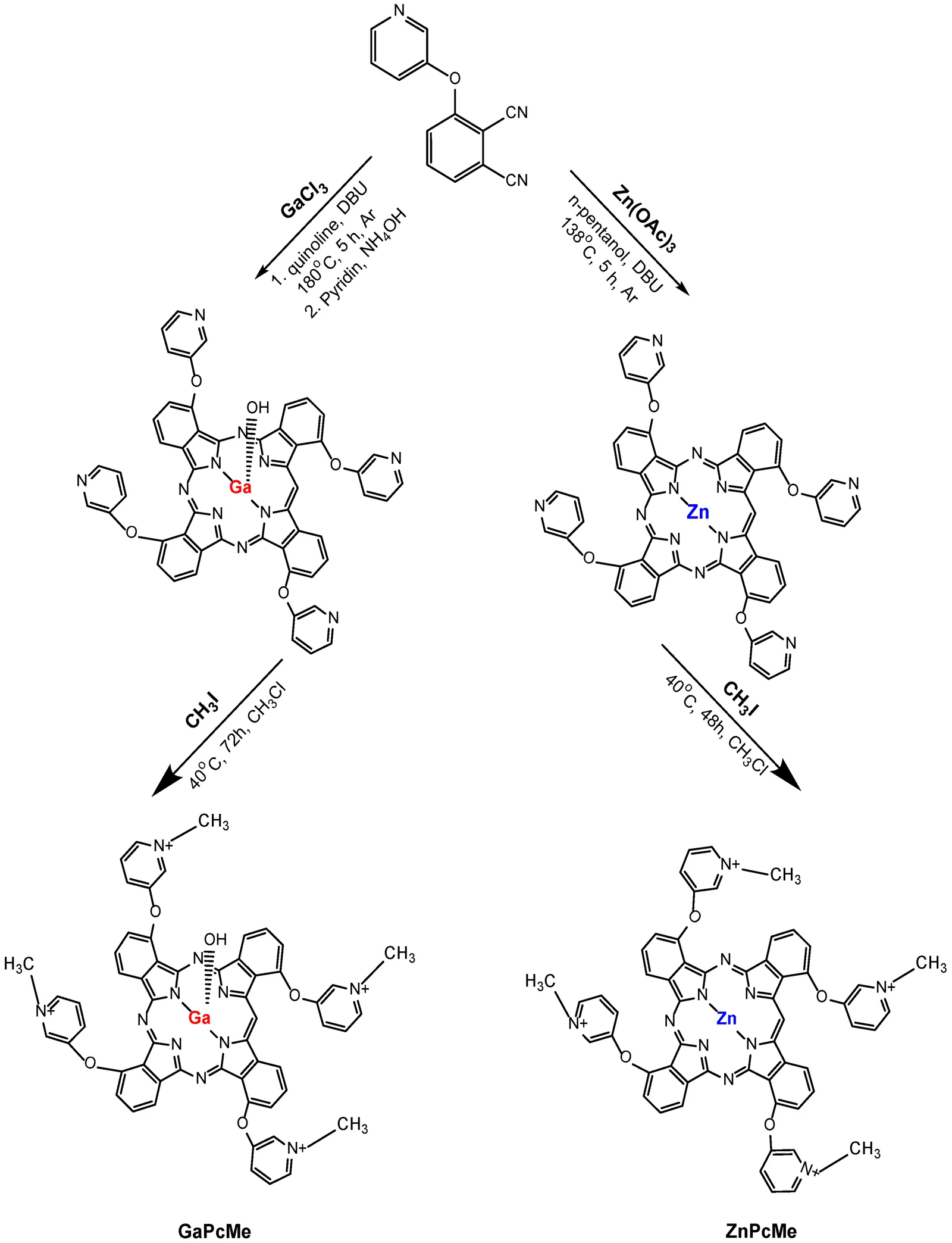
Synthesis of the phthalocyanines GaPcMe and ZnPcMe for a PDI study on HCoV-OC43.

Two non-peripheral tetra-methylpyridiloxy-substituted phthalocyanine complexes of gallium (GaPcMe) and zinc (ZnPcMe) were prepared following similar synthetic pathways to those previously reported [15,16]. The synthesis of both phthalocyanine complexes is shown in Scheme 1. 

**Figure 1:**
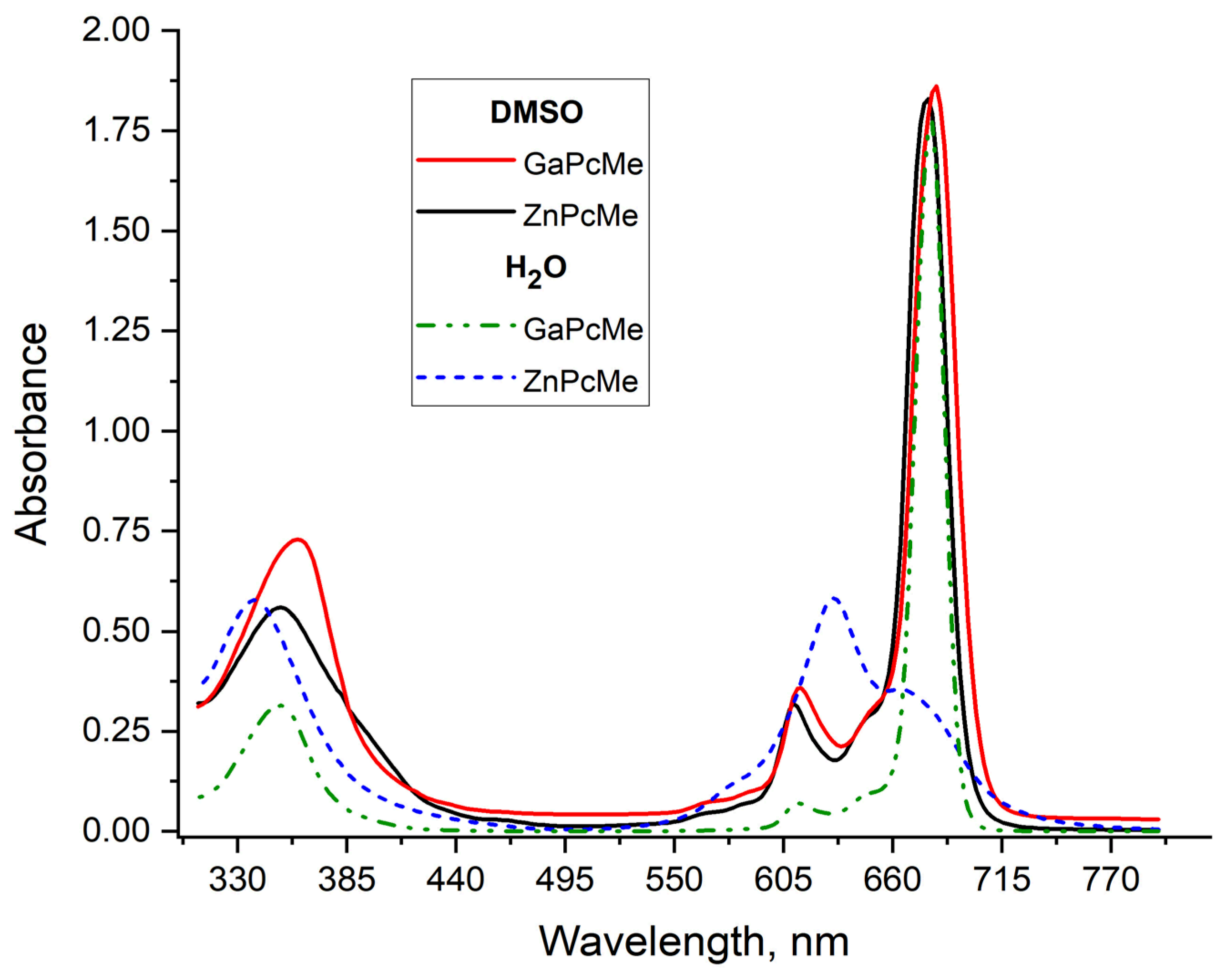
Absorption spectra of phthalocyanines in dimethyl sulfoxide and water (10^−5^ M).

Before in vitro experiments, two stock solutions of 2 mM GaPcMe and ZnPcMe were prepared in dimethyl sulfoxide (Merck Bulgaria EAD, Sofia, Bulgaria). The solutions were stored in closed vials covered with aluminum foil. The serial dilutions were made in sterile 1x phosphate-buffered saline (PBS) to ensure the DMSO content was below 5% of the total volume. The exact concentrations of the compounds were determined from absorption spectra recorded with a PerkinElmer Lambda 25 UV-vis spectrophotometer (PerkinElmer, Baden-Württemberg, Germany). The absorption spectra in DMSO show that both compounds are in a photoactive monomeric state (Figure 1). Still, in aqueous solution, ZnPcMe aggregated. All solvents and solids for synthesis and photophysicochemical studies were purchased from Sigma-Aldrich (Schnelldorf, Germany).

### 2.2. Measurement of Singlet Oxygen Quantum Yields (Φ_Δ_)

Using unsubstituted ZnPc as the reference material, singlet oxygen measurements were carried out as a 1,3-diphenylisobenzofuran (DPBF) oxidation reaction in atmospheric air (no oxygen bubbling). Usually, a 3 mL volume of the appropriate phthalocyanine (ZnPcMe or GaPcMe) containing the singlet-oxygen quencher (DPBF) was irradiated in the Q-band region at a concentration of 1 × 10^−5^ M. The Φ_Δ_ values of the phthalocyanine complexes under study were calculated using the following equation:

(1) $$\Phi_{∆}\;=\;{\Phi_{∆}^{Std}}_{\cdot }\frac{R\cdot I_{abs}^{Std}}{R_{Std}\cdot I_{abs}}$$

where Φ_Δ_^Std^ denotes the standard compound’s singlet-oxygen quantum yield. As a standard, unsubstituted ZnPc (Φ_Δ_^Std^ = 0.67 in DMSO) was used. The DPBF photo-bleaching rates in the presence of the investigating samples (ZnPcMe and GaPcMe) and the standard are denoted by R and R^Std^, respectively. The rates at which the samples and standards absorb light are indicated by I_abs_ and I_Δ_^Std^, respectively. The concentration of the quencher (DPBF) was kept below 30 µM to prevent chain reactions caused by DPBF in the presence of singlet oxygen. The stock solutions of ZnPcMe or GaPcMe at 10 µM, together with the molecular probe DPBF, were mixed in a cuvette and exposed to a 660 nm LED. Photooxidation of DPBF was followed by absorbance at 417 nm for approximately three to four minutes. 

### 2.3. Cells

Vero E6 (isolated from the kidney of an African green monkey) cells were provided by the National Bank for Industrial Microorganisms and Cell Cultures, Sofia, Bulgaria. The cells were cultured in DMEM growth medium (Gibco, Grand Island, NY, USA) containing 10% fetal bovine serum (Gibco, Grand Island, NY, USA), supplemented with 10 mM HEPES buffer (AppliChem GmbH, Darmstadt, Germany) and antibiotics (penicillin 100 IU/mL, streptomycin 0.1 mg/mL). Incubation was performed in an incubator (HERA cell 150, Heraeus, Hanau, Germany) at 37 °C and a 5% CO_2_ atmosphere. For in vitro experiments, the cells were plated in cell culture plates. After 24 h of incubation under the indicated conditions, the cells were treated with ZnPcMe and GaPcMe under study and illuminated according to the experimental setup.

### 2.4. Viruses

Human Coronavirus OC43 (HCoV-OC43) (ATCC: VR-1558) (American Type Culture Collection, Manassas, VA, USA) strain was propagated in Vero E6 cells in DMEM medium supplemented with 2% fetal bovine serum, 100 U/mL penicillin, and 100 μg/mL streptomycin. Cells were lysed 5 days after infection by 2 freeze-and-thaw cycles and the virus was titrated according to the Reed and Muench formula [17]. Virus and mock aliquots were stored at −80 °C. The infectious titer of the stock virus was 10^6.5^CCID_50_/mL.

### 2.5. Light Source

The used light source was a light-emitting diode (LED) with an irradiation spectrum with a maximum of 660 nm (ELO Ltd., Sofia, Bulgaria) which overlaps the absorption spectra of the applied phthalocyanines (Scheme 1). The samples were irradiated with a constant fluence rate of 50 mW/cm^2^ and a light dose of 12 J/cm^2^. The fluence rate was constant as measured with a power meter PM 100D with a sensor S120VC (Thorlabs Inc., North Newton, KS, USA).

### 2.6. Reference Compound

Remdesivir (GS-5734, RDV, REM, Veklury^®^) (Gilead Science, Dublin, Ireland) was initially dissolved in double-distilled water to a concentration of 150 mg/mL and then diluted in DMEM nutrient medium to the required concentrations.

### 2.7. Irradiated and Cytotoxicity Assay 

The cytotoxicity of the tested photosensitizers was determined without irradiation (I) dark toxicity and after irradiation (II) phototoxicity. The methodology is described in detail in our previous study [18]. Briefly, a monolayer cell culture of Vero E6 cells was inoculated with decreasing dilutions of the respective photosensitizer, after which the cells were incubated in the dark in a humidified atmosphere at 37 °C and 5% CO_2_. After 120 h of incubation, the cells were washed and the Neutral Red Uptake Assay was used to determine cell viability [19]. The 50% cytotoxic concentration (CC_50 Dark)_ was defined as the concentration of the applied compound that reduced cell viability by 50% compared to untreated controls. Each sample was tested in triplicate, with four wells for cell culture per sample. The maximum tolerable concentration (MTC _Dark_) was determined by the concentration at which the compound does not affect the cell monolayer. 

The phototoxicity of the phthalocyanine complexes was determined using a similar experimental setup, except that the samples were irradiated immediately after photosensitizer inoculation for 20 min at room temperature. The cytotoxic concentration of 50% under irradiation (CC_50 LED 660 nm_) and the maximum tolerated concentration under irradiation (MTC _LED 660 nm_) were determined. The ratio of the two 50% cytotoxic concentrations (in the dark and under irradiation) for each phthalocyanine complex indicates the photoinhibition factor (PIF). The PIF was determined according to Equation (1):

PIF = CC_50 Dark_/CC_50 LED 660 nm_ (2)

### 2.8. Determination of Infectious Viral Titers

A confluent monolayer of Vero E6 cells was infected with 0.1 mL of virus suspension using the final dilution method [17]. After 2 h of adsorption, the unadsorbed virus was removed, and 0.1 mL/well of maintenance medium was added. The plates were incubated for 120 h at 37 °C and 5% CO_2_ in a HERA cell 150 CO_2_ incubator (Radobio Scientific Co., Ltd., Shanghai, China). Two types of controls were also cultured in parallel—a cell control from uninfected cells, as well as the virus control—cells infected with the maximum concentration of virus and demonstrating the maximum cytopathic effect. The infectious virus titer was determined by determining the cytopathic effect (CPE). Visually detected CPE was confirmed by neutral red staining. Viral titers are expressed as lg IU (infectious units) (CCID_50_)/0.1 mL.

### 2.9. Photodynamic Inactivation

The experimental setups for the in vitro treatment of HCoV-OC43, including phthalocyanine concentrations and irradiation doses under the established treatment conditions, are presented in Table 1. In all experiments for PDI of HCoV-OC43, three types of virus controls were used: (I) a virus control not treated with photosensitizer and not irradiated; (II) a virus control that was irradiated but not treated with the photosensitizer; and (III) a virus control that was not irradiated but treated with the photosensitizer.

#### 2.9.1. Effect on Extracellular Virions

Samples were prepared containing an equal ratio of virus suspension (1:1) (10^5^ CCID_50_) and the applied complex at its maximum tolerated concentration (MTC _LED 660 nm_). The samples were irradiated for different periods of time (5, 10, 15 and 20 min) at room temperature. The viral titers were then determined and the residual viral infectivity in the sample was determined. The photoinactivation activity was expressed as the difference between the infectious viral titer of the tested sample and the controls (Δlg), as summarized in Table 1.

#### 2.9.2. Effect on Viral Adsorption

Two experimental setups were used to conduct this experiment.

##### 2.9.2.1. First Experimental Setting

The methodology is described in detail in our previous study [18]. Briefly, monolayer cell culture of Vero E6 cells was pre-cooled to 4 °C and inoculated simultaneously with 10^5^ CCID_50_ of HCoV-OC43 and the tested phthalocyanine complexes in their MTC _LED 660 nm_ (presented in Table 1). In this experimental setup, the samples were irradiated for different durations to assess virus adsorption. After incubation, the monolayer was washed with PBS to remove residual photosensitizers and unattached virus. Then they were covered with maintenance medium and incubated at 37 °C for 48 h. After triple freezing and thawing, the infectious viral titer of each sample (and control) was determined by the final-dilution method, and ∆lg values were calculated. Each sample was prepared in quadruplicate (Table 1).

##### 2.9.2.2. Second Experimental Setting

In the second experimental methodology, the irradiation intervals were different. In the first sample, the irradiation time started from the moment of inoculation of the virus to the cell monolayer in the presence of the photosensitizer and continued for the first 15 min of virus adsorption. In the second sample, irradiation began at 16 to 30 min of adsorption; in the third sample at 31 to 45 min; and in the fourth sample from 46 to 60 min of virus adsorption. Viral titers in the samples were determined and ∆lg was calculated compared to viral controls (Table 1).

#### 2.9.3. Effect on the Viral Replicative Cycle 

To determine the influence of the tested photosensitizers on viral replication in host cells, a cytopathic effect inhibition (CPE) assay was used. Remdesivir (REM) was used in parallel as a control. The methodology has been presented in detail [18]. Briefly, a monolayer of Vero E6 cells was infected with 100 viral infectious doses (CCID_50_) in 0.1 mL. After viral adsorption, the unattached viruses were removed, and the phthalocyanines were added at different concentrations. This was followed by 20 min of irradiation. After removal of the non-attached compound, the cells were incubated for 48 h at 37 °C (Table 1). CPE was determined using a neutral red absorbance assay. The percentage of CPE inhibition for each concentration of the photosensitizer tested was calculated using the following Formula (3):

% CPE = [OD_test sample_ − OD_virus control_]/[OD_toxicity control_ − OD_virus control_] × 100, (3)

where OD_test sample_ is the optical density (OD) of the samples inoculated with the virus and treated with the photosensitizer, OD_virus control_—inoculated with the virus but without photosensitizer in the medium, and OD_toxicity control_—samples without virus but treated with photosensitizer. 50% photoinactivating concentration (PIC_50_) is defined as the concentration of the tested photosensitizers that inhibits 50% of the viral replication compared to the virus control. The determination of PIC_50_ is performed under dark conditions (PIC_50 Dark_) and under irradiation with PIC_50 LED 660 nm_. Photoinactivating index (PII), which represents the selectivity of the tested photosensitizers, is calculated from the ratio (3):

CC_50 LED 660 nm/_PIC_50 LED 660 nm_. (4)

### 2.10. In Silico Simulations

Crystal structures of targets were downloaded from the Protein Data Bank (PDB). The HCoV-OC43 main protease 3CLpro (Nsp5, PDB 9PAM) was included to provide structural context directly related to the virus used in the in vitro experiments. In addition, homologous SARS-CoV-2 proteins Nsp5 (PDB 7QBB), Nsp12 (PDB 7AAP), M protein (PDB 7VGR), S protein (PDB 6VSB), and human ACE2 (PDB 1R42) were used for docking [20,21,22,23,24]. These proteins were selected because they are relevant to coronavirus replication, assembly, or host–cell entry, and the computational analysis was intended to generate mechanistic hypotheses rather than to directly model the HCoV-OC43 experimental system.

These structures were prepared for docking by removing non-inherent water molecules. In addition, polar hydrogens were added, and Gasteiger charges were assigned. Compound structures were drawn using the ChemSketch program. Similarly, compounds were prepared for docking by adding polar hydrogens and assigning Gasteiger charges. Grid boxes for docking were specified based on co-crystal ligands. Blind docking was undertaken for the structure without a co-crystal ligand. Molecular docking was performed using AutoDock Vina (1.2.7) [25,26]. Docking results were visualized and analyzed using Biovia Discovery Studio 2025 (Dassault Systèmes, San Diego, CA, USA).

Relative stabilities of complexes procured from the docking study were compared using MD simulation. Compound topologies were generated through the CGenFF server (MacKerell laboratory, SilcsBio, Baltimore, MD, USA) [27]. Protein structure topology files were generated using the GROMACS 2024 software package and mapped to the CHARMM36 force field. Prepared structures were placed in a dodecahedral simulation box. A minimum distance of 1.0 nm was maintained between the protein atoms and the box edges. The system was solvated using the CHARMM-modified TIP3P water model. System neutralization to mimic physiological ionic strength was achieved by substituting the counterions with those from NaCl. Energy minimization was performed using the steepest descent algorithm. System equilibration was performed using the canonical (NVT) and isobaric-isothermal (NPT) ensembles. MD simulations were performed in the NPT ensemble for 200 ns using GROMACS 2024 [28]. The temperature was sustained at 300 K using a thermostat. Possible general stability, residual level changes, and compactness-related alteration metrics were calculated over 200 ns of production. Then, an analysis was undertaken by drawing related plots using QtGrace (Qt-based Grace implementation for Windows) [29]. It should be noted that the MD simulations were conducted as single trajectories without independent replicas. Therefore, the observed trends should be interpreted as indicative rather than conclusive evidence of complex stability.

### 2.11. Statistical Analysis

Data on the cytotoxicity of photosensitizers and antiviral effects were analyzed statistically. The values of CC_50_, IC_50_, PIC_50,_ and Δlg were presented as mean ± SD. The effects of each photosensitizer on extracellular virions and the stages of their adsorption were compared with the untreated viral control and statistically analyzed using Student’s *t*-test. When using Student’s *t*-test, *p*-values of <0.05 were considered significant. Data were analyzed by GraphPad Prism 8 Software (GraphPad, San Diego, CA, USA).

## 3. Results

**Figure 2:**
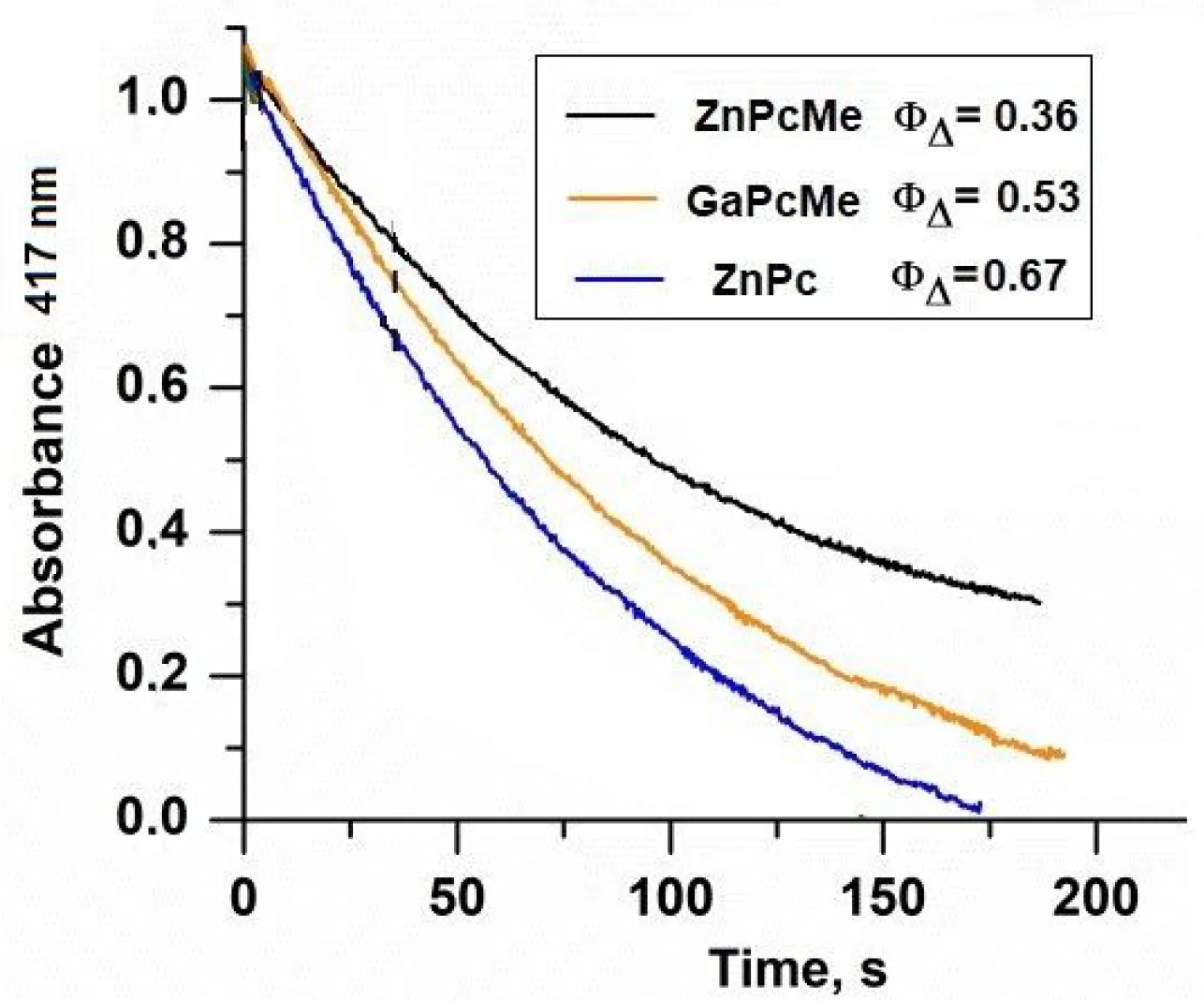
Quenching of singlet oxygen by DPBF during 660 nm irradiation of the studied phthalocyanines.

Singlet oxygen (^1^O_2_), a major cytotoxic molecule in PDT, is generated by the photochemical interaction between the excited triplet state of PS and the surrounding oxygen. The efficiency of this generation depends on the efficient energy transfer from the excited triplet PS-state to the ground triplet oxygen state. The singlet oxygen production upon 660 nm LED irradiation was determined. A Type II PDT reaction mechanism was assessed. Multiple factors, including the singlet-oxygen quenching capabilities of the substituents’ size and position and the solvent, which affects the efficiency of energy transfer between PS and the surrounding oxygen, influence the process of PDT reactions. Figure 2 shows the changes in the absorption due to oxidation of the probe (DPBF). When compared to the central metal atom, gallium complexes have a higher ΦΔ value than zinc complexes because of the heavy atom effect [30,31].

The presence of substituents and different metal ions at the center was observed to affect singlet oxygen production in the studied phthalocyanines (Figure 2). The quantum yield was determined using an indirect method based on the chemical quenching of a molecular probe (DPBF). The absorbance of the probe at 417 nm decreased with 660 nm irradiation for both complexes. The singlet oxygen yields were lower for GaPcMe and ZnPcMe than for their respective unsubstituted ZnPc counterparts. The intensity of the Q band of the complexes remained stable during DPBF oxidation, indicating that the compounds were not degraded by singlet oxygen generation. 

**Figure 3:**
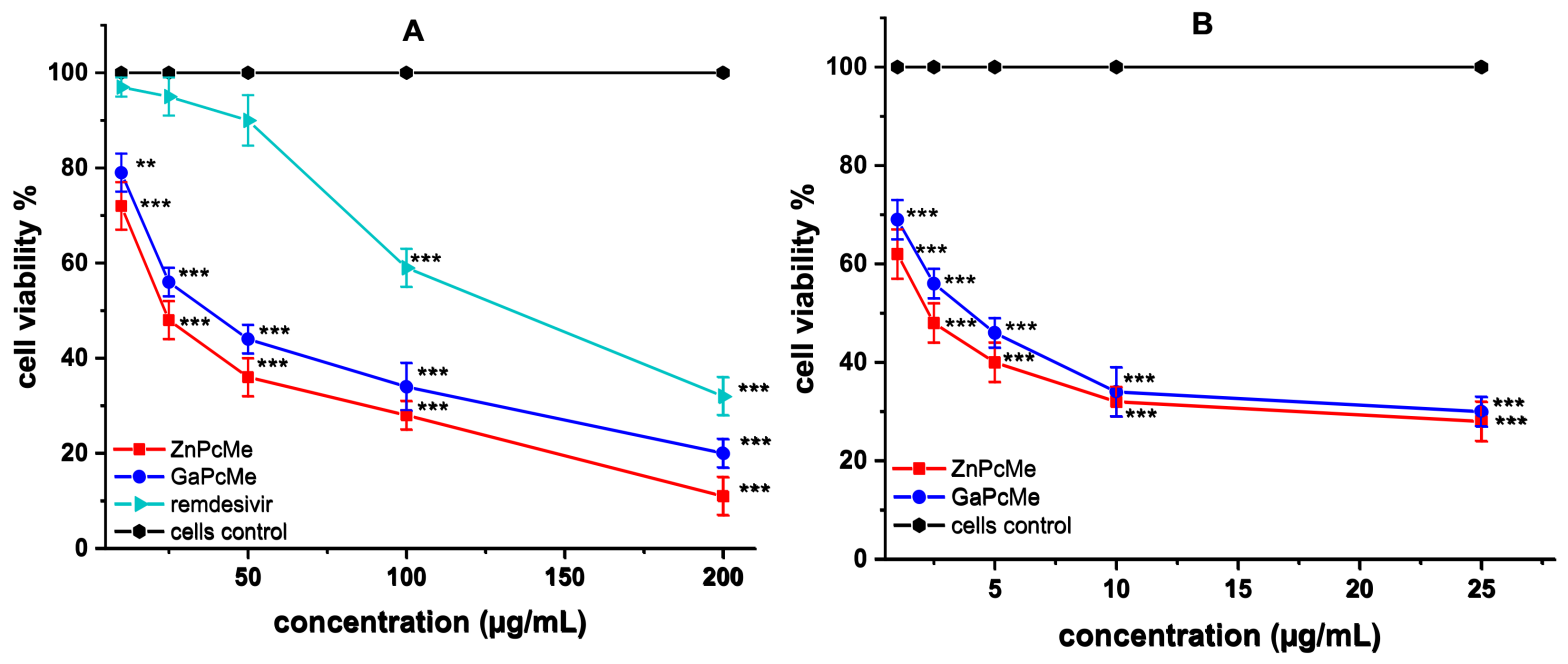
Cytotoxicity of phthalocyanines (ZnPcMe and GaPcMe) against Vero E6 cells determined: (**A**) in the dark and (**B**) upon LED-660 nm irradiation. ** *p* ˂ 0.01, comparing the value for each substance with the cell control; *** *p* ˂ 0.001, comparing the value for each substance with the cell control, Student’s *t*-test.

To avoid dark- and light-induced cytotoxicity of the studied phthalocyanines, ZnPcMe and GaPcMe, on host cells, their effects were initially assessed on the Vero E6 cell monolayer. The experiments were carried out in the dark and at 20 min in LED-660 nm light after the application of ZnPcMe and GaPcMe. The study showed similar cytotoxicity of the two compounds across both regimes—dark and light. However, in the case of ZnPcMe, slightly higher values of dark and phototoxicity were observed. The cytotoxic concentrations that reduced cell monolayer viability by 50% in the dark (CC_50_ Dark) and under irradiation (CC_50 LED 660 nm_) were determined (Figure 3). The results are summarized in Table 2. The calculated photoinhibition factor (PIF) is the ratio of the CC_50_ in the dark to the CC_50 LED 660 nm_ after irradiation. As seen, ZnPcMe showed a higher PIF (PIF = 11.04) than GaPcMe, which was evaluated with a slightly lower value (PIF = 8.2). The so-called maximum tolerable concentration of a photosensitizer was also determined. This is a particular concentration at which there is no visible damage to the cell monolayer in the dark (MTC _Dark_) as well as under light conditions (MTC _LED 660 nm_). Both compounds showed the same MTC values, with the higher value in the dark (MTC _Dark_ = 5.0 µg/mL) and the expected lower value in the light (MTC _LED 660 nm_ = 0.5 µg/mL), as shown in Table 2. Settling the MTC after LED-660 nm is important, since the antiviral experiments were carried out assuming this concentration to be a limitation.

**Figure 4:**
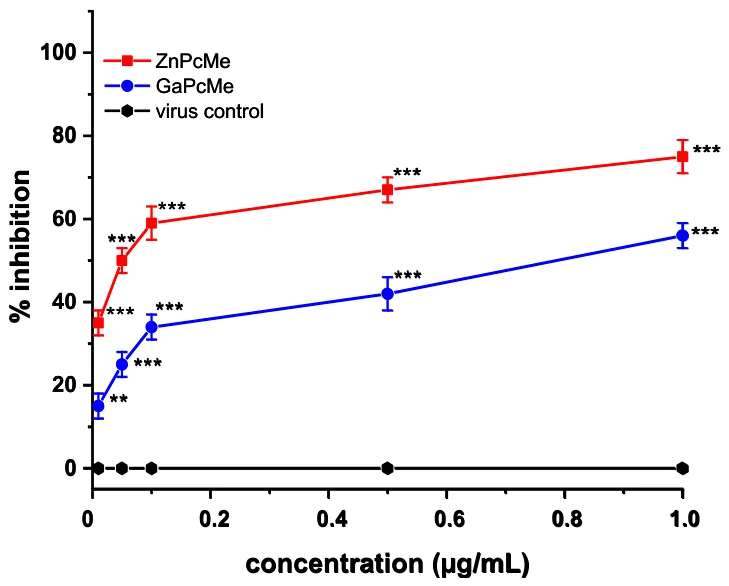
Photodynamic inactivation of the HCoV-OC43 replication cycle. ** *p* ˂ 0.01, comparing the value for each substance with the virus control; *** *p* ˂ 0.001, comparing the value for each substance with the virus control, Student’s *t*-test.

Following the experiments on the cell monolayer, the infectivity of HCoV-OC43 at different stages of its reproduction was monitored after incubation with ZnPcMe and GaPcMe. A general scheme of the antiviral experiments together with the irradiation intervals that were applied to the virus, in dependence on the occurrence of viral infection (Table 1, Section 2.9). The inhibitory effect of PDI on HCoV-OC43 replication in host cells was evaluated in the dark and under irradiation for 20 min. after viral adsorption. The previously established non-toxic concentrations of the photosensitizers were used. After the viral adsorption period, the unadsorbed viral particles were removed, ensuring that subsequent irradiation was performed only on viral particles that had penetrated the sensitive cells. Under dark conditions, no inhibitory effect was observed. Upon illumination, the photosensitizers were activated and demonstrated a photodynamic antiviral effect. Their PIC_50_, at which 50% of the viral yield was inhibited, was determined relative to the irradiated virus control (Figure 3). The selectivity of the antiviral effect was determined by calculating its photoinactivation index (PII). ZnPcMe exhibited remarkable photosensitizing activity, as evidenced by a PII of 44.0. The same parameter for GaPcMe was calculated to be almost two times lower (23.3) but comparably optimal (Figure 4, Table 2).

**Figure 5:**
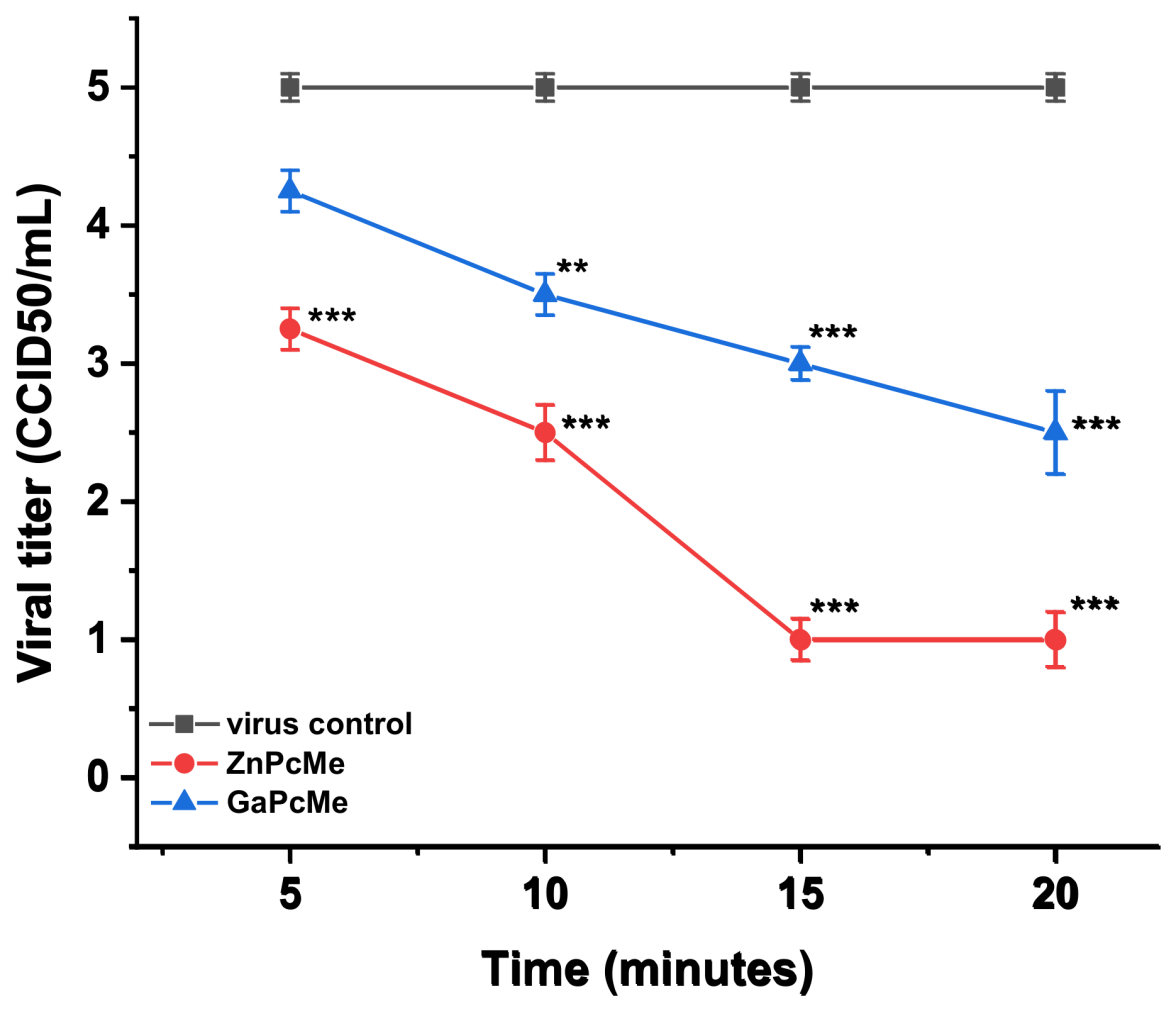
Photodynamically activated virucidal activity of ZnPcMe and GaPcMe against HCoV-OC43 virions. Phthalocyanine complexes were applied in their MTC _LED 660 nm_, which was previously determined (Table 2); ** *p* ˂ 0.01, comparing the value for each substance with the virus control; *** *p* ˂ 0.001, comparing the value for each substance with the virus control, Student’s *t*-test.

The effect of PDI on extracellular virions before they came into contact with susceptible cells (Vero E6) was also investigated. In Table 1, the irradiation period is in accordance with Section 2.9.1. The virus was incubated with ZnPcMe or GaPcMe for varying durations in the dark and under irradiation, in the absence of cells. The titer of the resulting samples was then determined to assess viral infectivity and to determine the Δlg parameter. Viral controls incubated with a PS, but not irradiated (dark control), and the control that was irradiated but did not contain PS (light control), showed no reduction in viral titer. The data suggest that: (1) in the dark, ZnPcMe and GaPcMe do not affect the viral particle and (2) the only irradiation of the virus does not lead to a reduction in its infectivity. ZnPcMe clearly inhibited viral particles within the first time interval, leading to a decrease in a viral titer of Δlg = 1.75. This effect is enhanced by increasing the light dose and the exposure time. At 15 and 20 min. of irradiation, the decrease in viral titer is Δlg = 4.0. Considering the light dose, GaPcMe at 5 min. exposure shows insignificant inhibition, but at 10 min. irradiation, the effect slightly increased to Δlg = 1.5. The longer the exposure, the more the effect is further enhanced. Although it becomes significant after 15 min of irradiation, a decrease in viral titer of Δlg = 2.5 was observed after 20 min. However, the effect was significantly weaker than that reported for ZnPcMe (Figure 5, Table 3).

**Figure 6:**
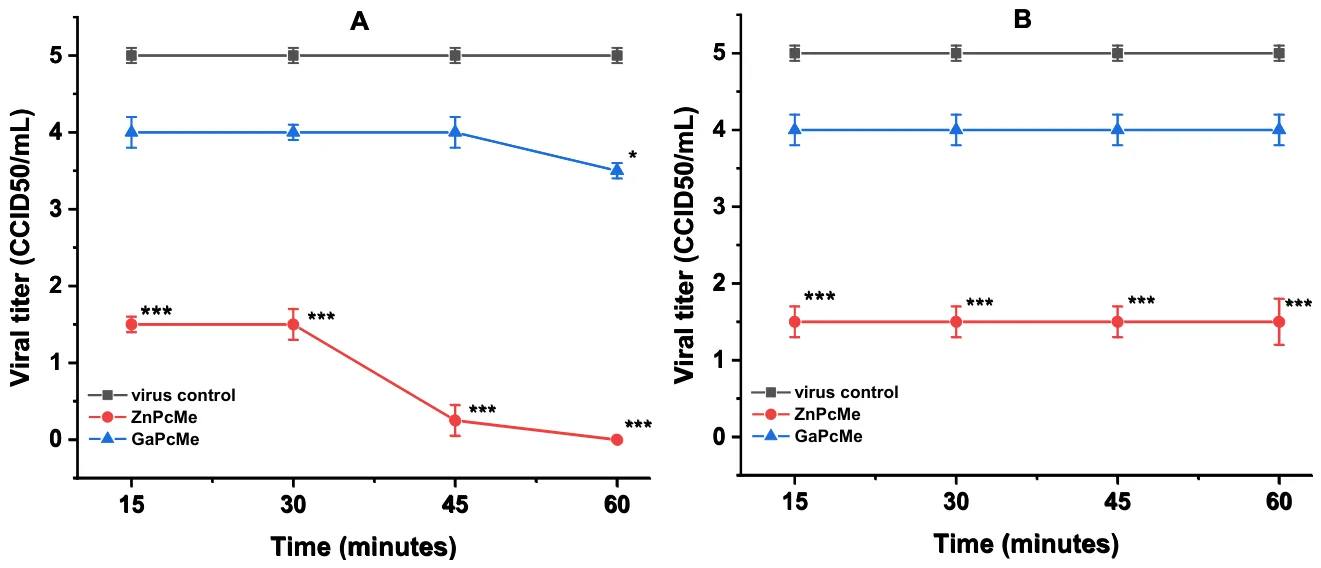
Photodynamic inactivation of the viral adsorption stage of HCoV-OC43 viral particles to Vero E6 cells. (**A**) Irradiation of different durations; (**B**) irradiation is of constant duration during different stages of viral adsorption. Phthalocyanine complexes were applied in their MTC _LED 660 nm_, which was previously determined (Table 2); *** *p* ˂ 0.001, comparing the value for each substance with the virus control, Student’s *t*-test.

The demonstrated photodynamic virucidal effects of both phthalocyanines motivated us to investigate their influence on viral adsorption onto sensitive Vero E6 cells. Two experimental setups were used, as presented in Table 1 (Section 2.9). In both incubation regimes, the virus and PSs are simultaneously added to the Vero E6 cell monolayer and irradiated for different durations during viral adsorption. In the first experimental setup, the irradiation time varies, starting at the beginning of the adsorption process. During the first 15 min of viral adsorption, ZnPcMe significantly inhibits the process, resulting in a decrease in viral titer of Δlg = 3.5. With increasing irradiation dose and exposure time, the effect is significantly enhanced, with 100% inhibition of viral adsorption achieved at 60 min of irradiation (Δlg = 5.0). Weak inhibition was observed for GaPcMe. The effect was weak for all irradiation intervals, with the strongest effect after 60 min of irradiation (Δlg = 1.5) (Figure 6A, Table 4).

In the second experimental setup, the irradiations were conducted at equal 15 min intervals, but at different times relative to the virus adsorption. The difference between the irradiation intervals in the two sets, First Experimental Setting Section and Second Experimental Setting Section, is presented in Table 1. The results showed that exposing ZnPcMe at different stages of viral adsorption to the same extent led to the same decrease in viral titer (Δlg = 3.5). In the case of GaPcMe, the PDI effect was very weak with Δlg = 1.0, regardless of the stage of viral adsorption at which the irradiation occurred (Figure 6B, Table 4).

The interaction potential of ZnPcMe and GaPcMe with HCoV-OC43 3CLpro (PDB 9PAM) and selected coronavirus-related targets was explored by molecular docking as a hypothesis-generating approach. Structural targets included SARS-CoV-2 Nsp5 (PDB 7QBB), Nsp12 (PDB 7AAP), M protein (PDB 7VGR), spike protein (PDB 6VSB), and human ACE2 (PDB 1R42). These SARS-CoV-2 proteins were chosen as structurally characterized homologues of key coronavirus replication and entry factors, allowing us to probe potential pan-coronaviral interaction patterns of the phthalocyanines beyond HCoV-OC43. In this context, the SARS-CoV-2 targets are used as structural surrogates to generate broader mechanistic hypotheses rather than to directly model the HCoV-OC43 experimental system. The predicted binding affinities of ZnPcMe and GaPcMe were compared with those of the respective co-crystallized ligands, where available, and with relevant antiviral reference compounds.

In general, both metal complexes showed comparable docking scores, with ZnPcMe displaying slightly more favorable predicted binding affinities than GaPcMe for most targets, except for Nsp12 (7AAP). The most favorable predicted binding affinities for both complexes were observed for Nsp12 (7AAP). On this basis, Nsp12 was selected for further molecular dynamics analysis, while the docking data were explicitly treated as preliminary, hypothesis-generating indications of possible target engagement rather than definitive evidence of the underlying mechanism.

**Figure 7:**
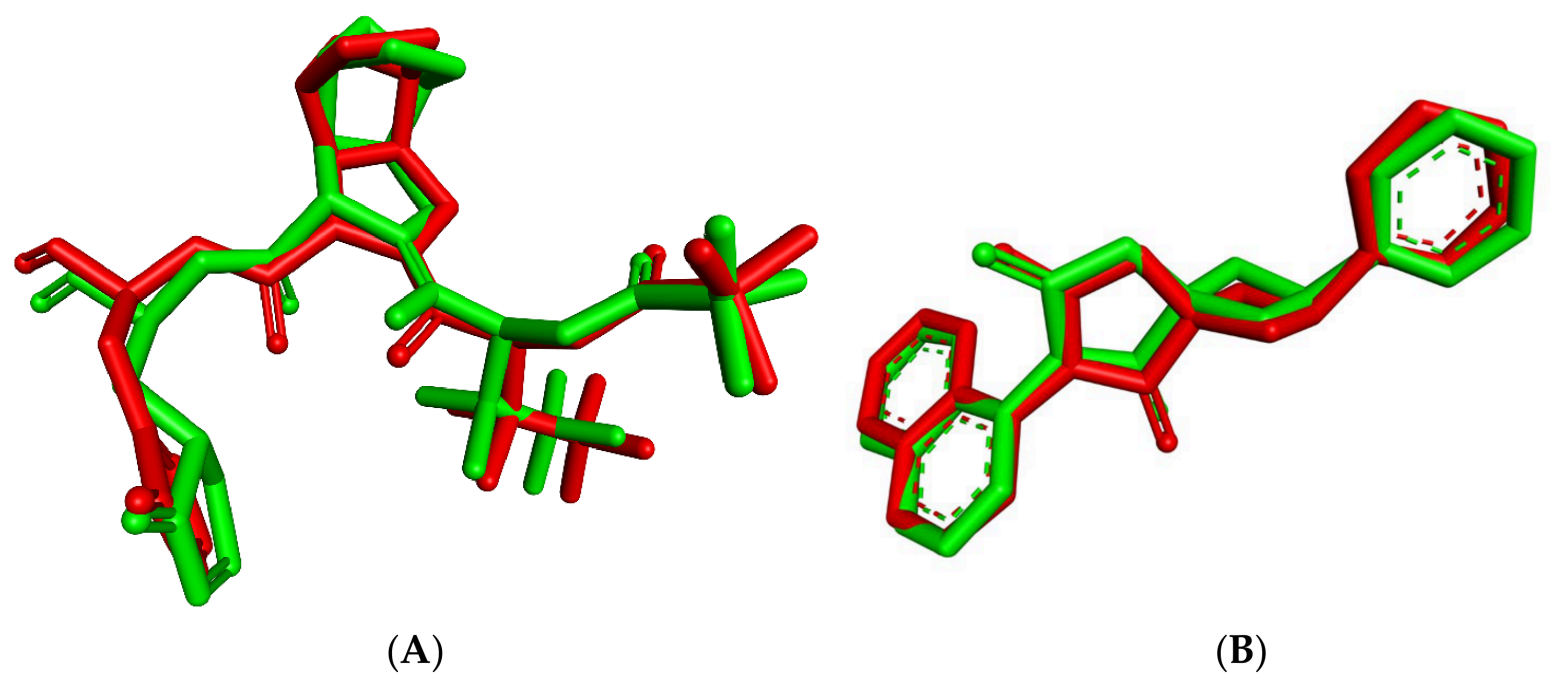
Superposition of the native crystal ligand conformation (green) and the redocked conformation (red) for the docking validation targets. (**A**): ALG-097655 for HCoV-OC43 3CLpro (PDB 9PAM). (**B**): the co-crystallized ligand for SARS-CoV-2 Nsp12 (PDB 7AAP). The close overlap between the native and redocked conformations indicates that the docking protocol can reproduce the experimentally observed ligand geometry.

Initially, the docking protocol was validated through redocking simulations for targets with available co-crystallized ligands. Redocking was carried out for HCoV-OC43 3CLpro (PDB 9PAM) and SARS-CoV-2 Nsp5 (PDB 7QBB). The root mean square deviation (RMSD) between the crystallographic and redocked ligand poses was calculated, yielding values of 0.5973 Å for ALG-097655 and 0.3637 Å for compound 18 (Figure 7). These low RMSD values indicate that the docking setup can reliably reproduce the experimental binding modes and is suitable for subsequent hypothesis-generating docking simulations.

Reliability of the molecular docking for the Zn and Ga phthalocyanines was ensured by implementing a rigorous parametrization strategy aimed at preserving the metal coordination environment. Phthalocyanines possess a rigid planar macrocyclic core with a central coordination cavity that restricts the geometrical freedom of the bound metal ions. For the Zn-derived complex, we utilized the standard AutoDock Vina force field parameters, which accurately represent the stable +2 oxidation state and its associated coordination geometry. In contrast, for the gallium (Ga^3+^) complex—an ion less common in standard docking libraries—we adopted a non-bonded model in conjunction with a rigid-core constraint. Ligand topologies were generated using the CGenFF. In the absence of explicit transition metal parameters, the CHARMM General Force Field (CGenFF) always assigns high penalty scores to metal–ligand bonds. We avoided this drawback by freezing the internal coordinates of the metal–ligand complex; the coordination geometry was pre-optimized with the Avogadro program and kept as a rigid body during docking. This approach rendered the high internal bond penalties irrelevant as the docking algorithm only sampled translational, rotational and non-core torsional degrees of freedom. Consequently, the Ga^3+^ ion was successfully described by exact van der Waals and electrostatic terms, free from the distortion induced by the internal bond limitations of the force field. The large hydrophobic surface area and concentrated positive charge of these cationic phthalocyanines necessitated the expansion of grid boxes to encompass extensive search spaces to attain reliable sampling without excessive conformational penalties.

**Figure 8:**
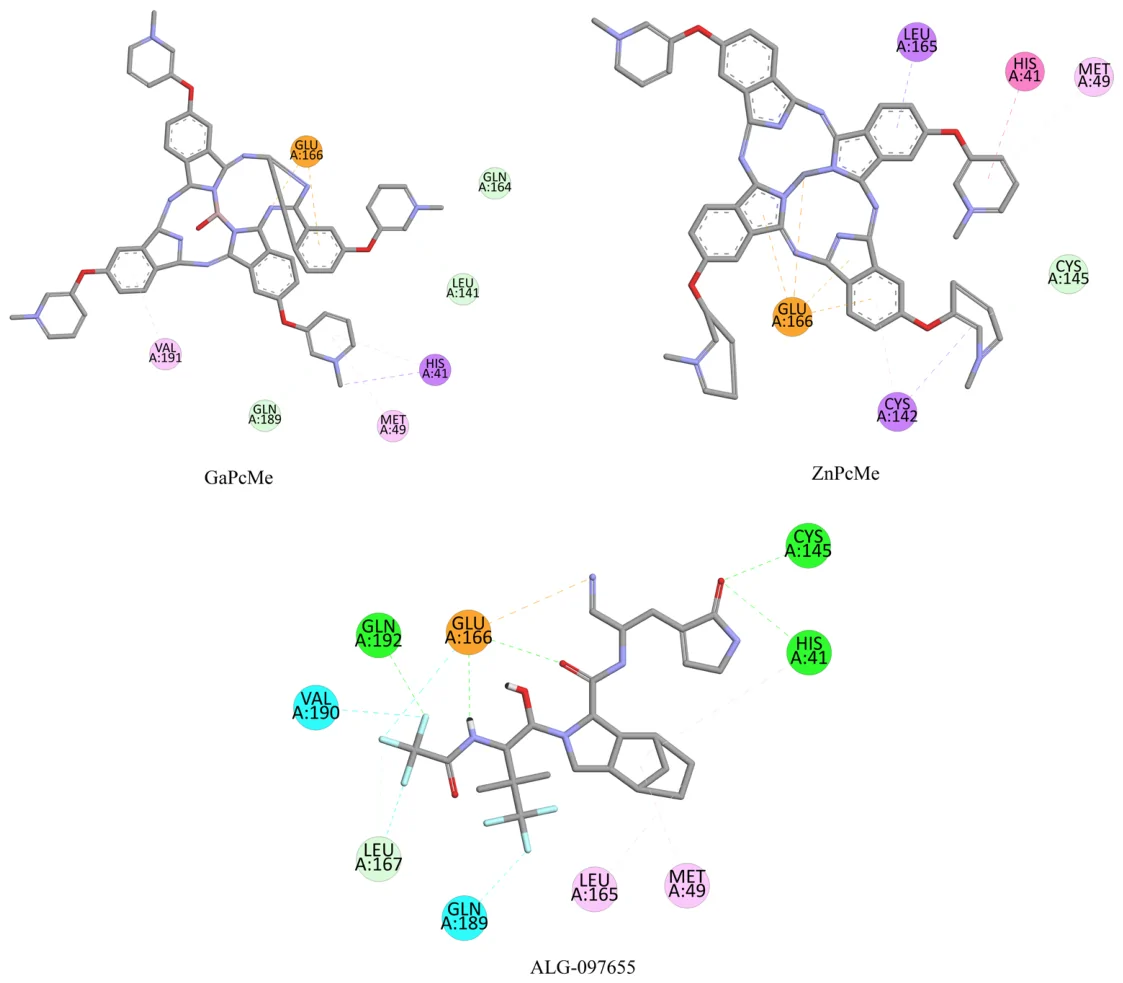
Binding profiles of ZnPcMe, GaPcMe, and ALG-097655 in their interaction with the HCoV-OC43 structure (9PAM).

**Figure 9:**
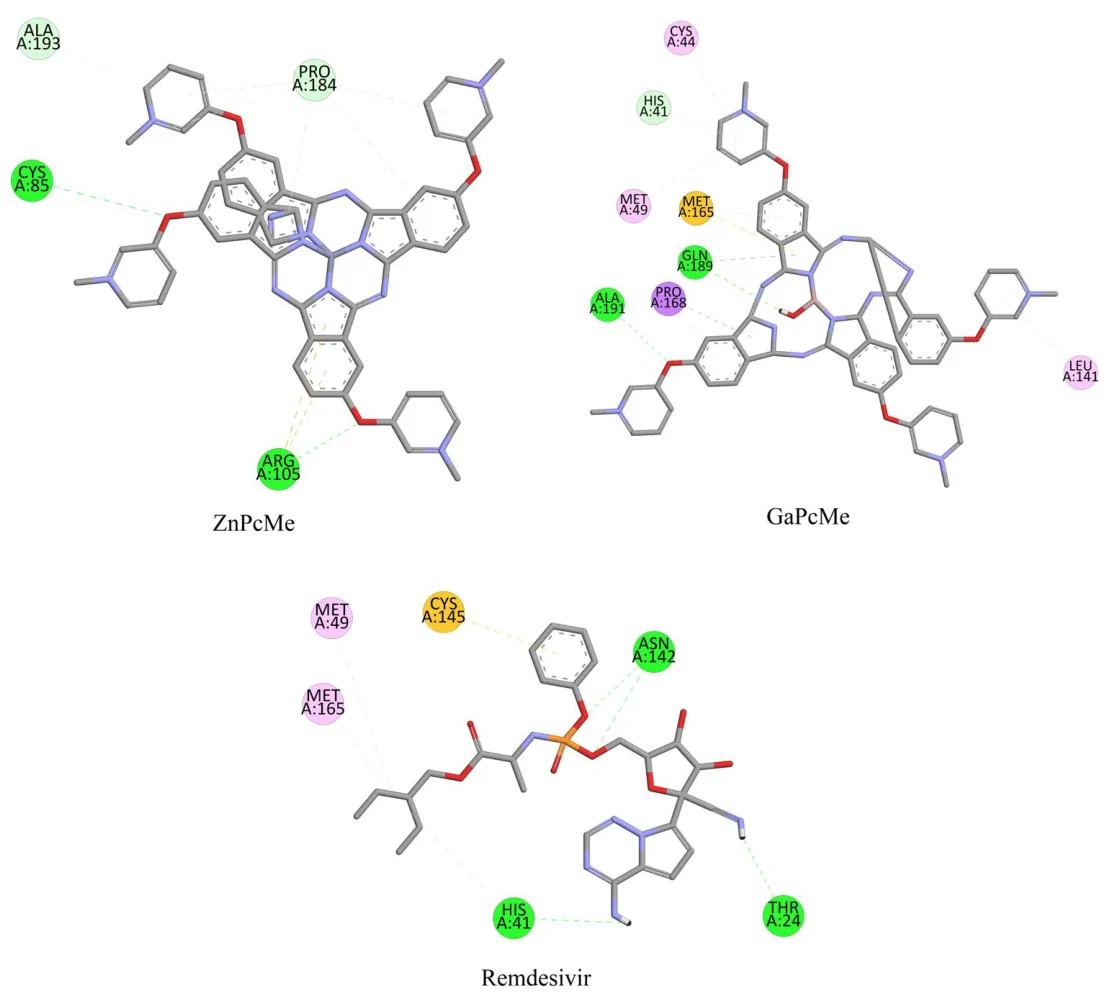
Binding type and residues of ZnPcMe, GaPcMe, and REM in their interaction with Nsp5 (7QBB).

**Figure 10:**
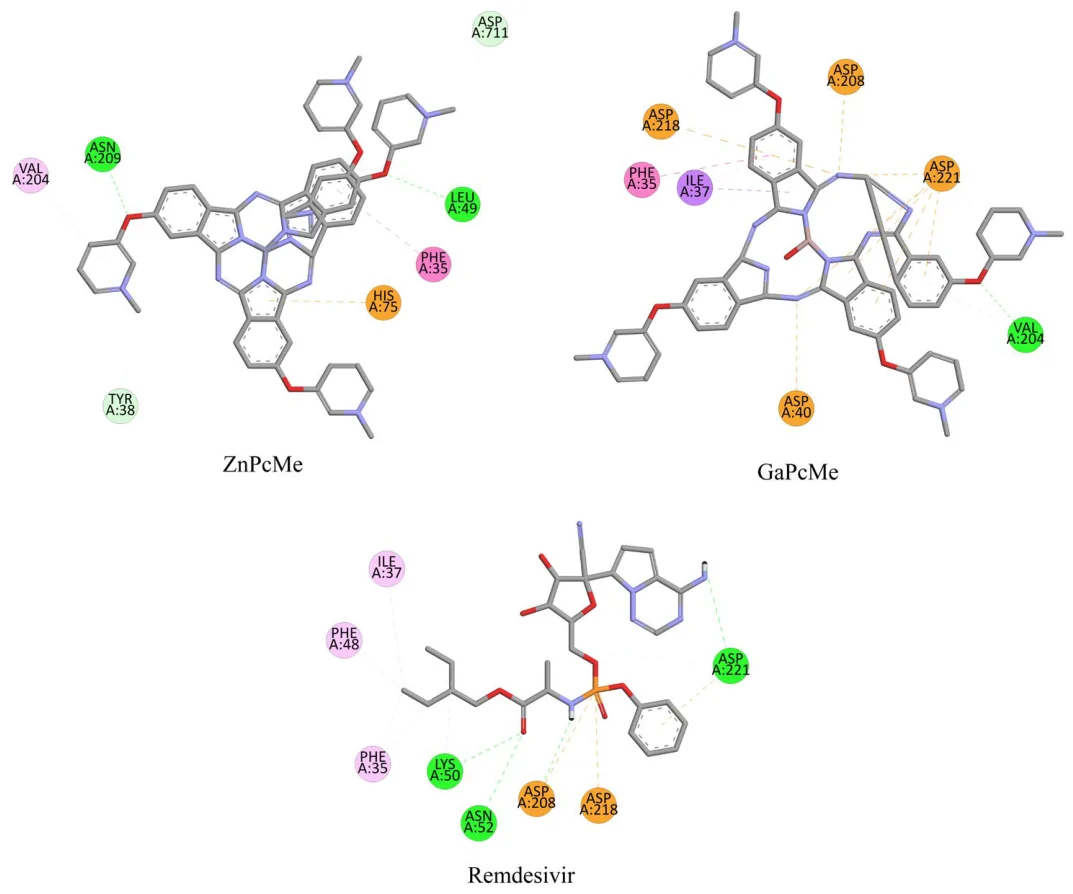
Binding type and residues of ZnPcMe, GaPcMe, and REM in their interaction with Nsp12 (7AAP).

**Figure 11:**
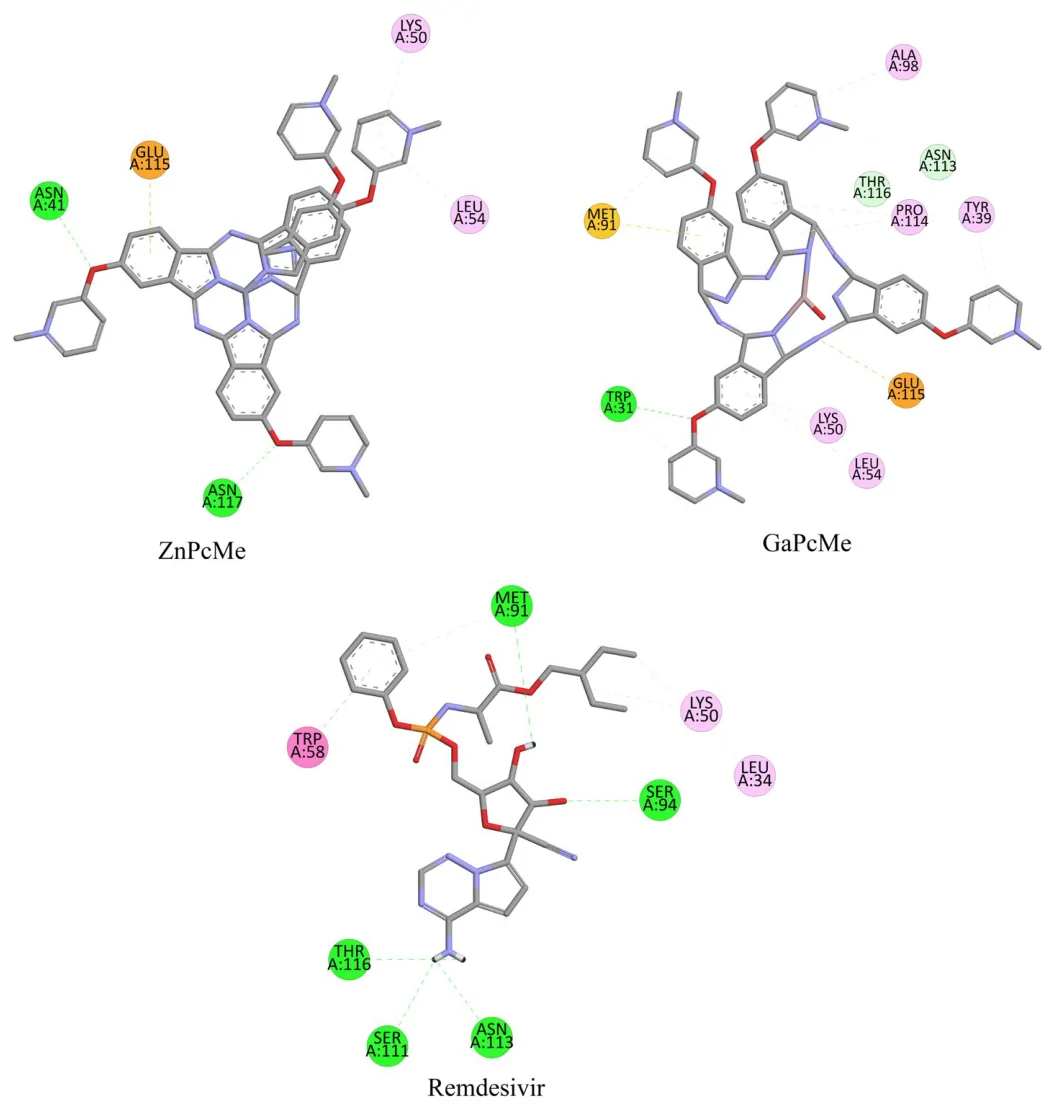
Binding type and residues of ZnPcMe, GaPcMe, and REM in their interaction with M protein (7VGR).

**Figure 12:**
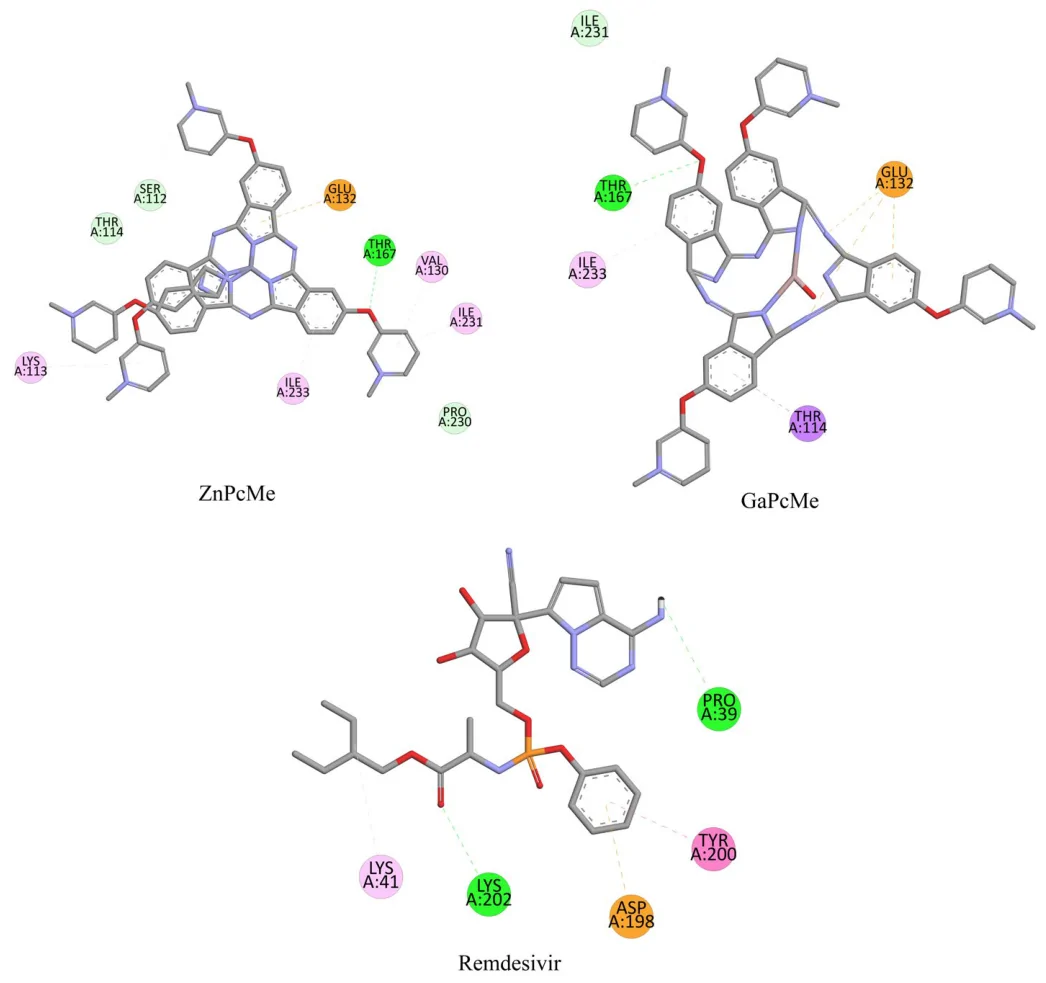
Binding type and residues of ZnPcMe, GaPcMe, and remdesivir in their interaction with S protein (6VSB).

**Figure 13:**
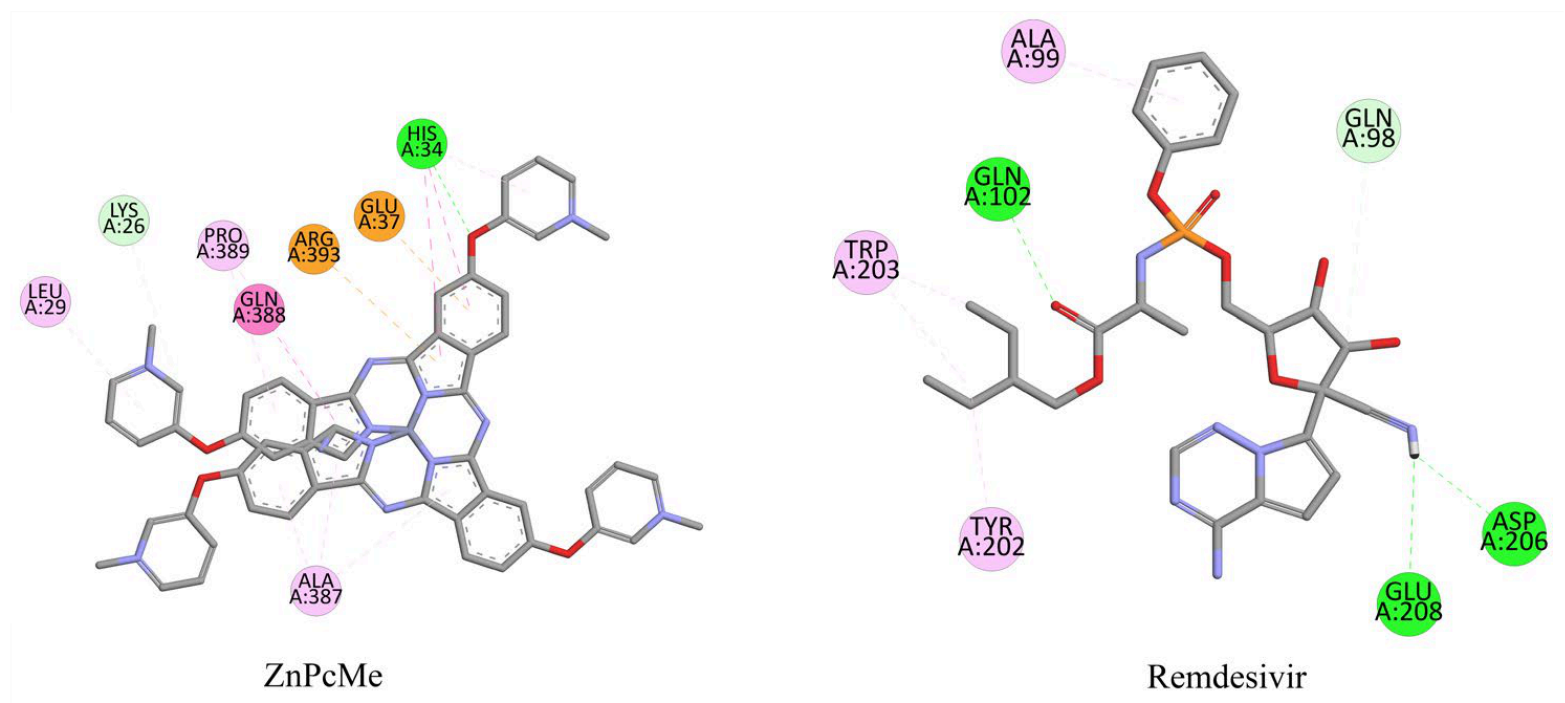
Binding type and residues of ZnPcMe and remdesivir in their interaction with ACE2 (1R42).

Metal complexes displayed diverse noncovalent interaction patterns with the target structures. In the HCoV-OC43 3CLpro target (PDB 9PAM), both GaPcMe and ZnPcMe showed strong predicted binding affinities and interacted with key catalytic-site residues, including His41, Met49, Cys145, and Glu166, with ZnPcMe exhibiting a slightly more favorable docking score than GaPcMe. The co-crystallized ligand ALG-097655 also formed interactions with the 9PAM active site, providing a useful structural reference for comparison. Overall, the metal complexes formed one or two conventional hydrogen bonds, whereas they established a larger number of nonconventional interactions, including hydrophobic, alkyl, and π-related contacts, across the target proteins (Table 5, Figure 8, Figure 9, Figure 10, Figure 11, Figure 12 and Figure 13). By contrast, remdesivir generally formed more conventional hydrogen bonds, but fewer nonconventional contacts than the metal complexes (Table 5, Figure 8, Figure 9, Figure 10, Figure 11, Figure 12 and Figure 13).

### MD Simulation

**Figure 14:**
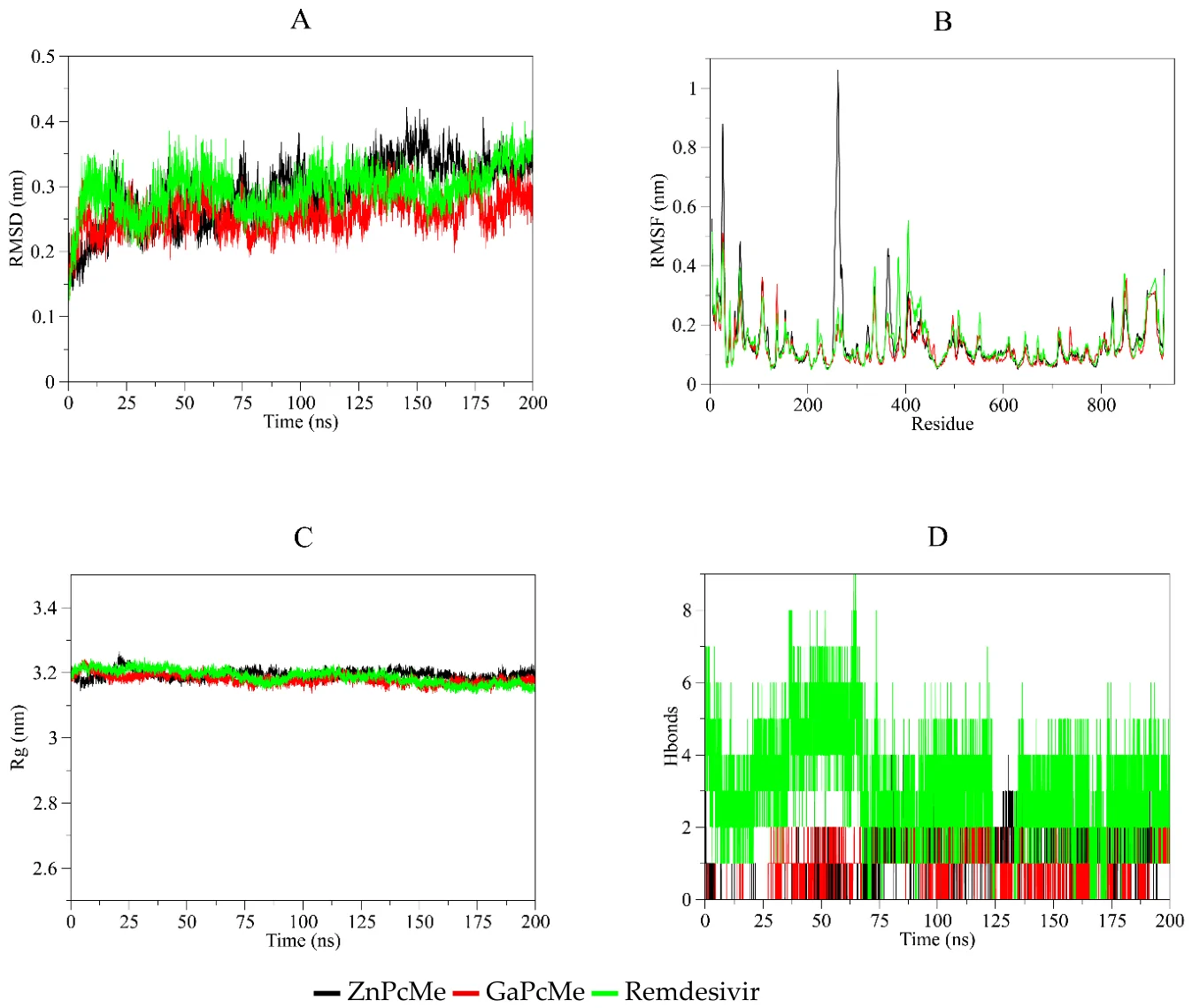
RMSD (**A**), RMSF (**B**), Rg (**C**), and hydrogen bond number (**D**) plots of compound complexes with Nsp12 (7AAP) structure.

Molecular docking suggested that both photosensitizers may interact with SARS-CoV-2 structural and nonstructural targets, with generally more favorable predicted binding affinities than remdesivir. Among the examined targets, Nsp12 (PDB 7AAP) yielded the most favorable docking scores for both metal complexes; therefore, this target was selected for subsequent molecular dynamics (MD) analysis. The MD simulations were performed to assess the stability of the GaPcMe, ZnPcMe, and reference complexes in the Nsp12 binding site. Overall, the metal complexes showed a stronger predicted binding tendency toward Nsp12, although these results should be interpreted as preliminary and hypothesis-generating rather than definitive evidence of target engagement. Root mean square deviation (RMSD), root mean square fluctuation (RMSF), radius of gyration (Rg), and hydrogen bond number plots were drawn based on MD simulation trajectories. RMSD plots of the three complexes (ZnPcMe, GaPcMe, and REM) were generally similar to each other. Differences between the three complexes were observed in narrow time intervals. In this regard, the ZnPcMe-containing complex gave slightly higher RMSD values in the 135–170 ns interval. On the other hand, the GaPcMe-containing complex showed lower RMSD values over some intervals, particularly during the last 23 ns (Figure 14A).

RMSF plots for three complexes were generated to assess their relative stability over the course of the simulation. In general, the three complexes gave close RMSF values. Together with this, the ZnPcMe-containing complex showed higher RMSF values in some intervals, notably the 22–30 and 255–266 residue intervals. Similarly, the REM-containing complex exhibited slightly higher RMSF values across some residue intervals (Figure 14B). Rg plots of the three complexes were also similar, with an average of nearly 3.19 nm. The ZnPcMe-containing complex gave slightly higher Rg values at some time intervals (Figure 14C). H-bond number plots of the compounds differed in both number and frequency. Compound ZnPcMe gave mainly two H-bonds, especially after 67 ns. Compound GaPcMe had mainly a single H-bond intercalated with two H-bonds. On the other hand, remdesivir gave mainly three- and four-H-bonds. In addition, it gave up to six H-bonds at some time intervals, including the 45–67 ns interval. In general, the stability of the H-bond number was low for all compounds (Figure 14D). 

## 4. Discussion

Aggregation in aqueous media is a disadvantage of phthalocyanines that negatively impacts their photochemical properties, since the resulting associates are not photoactive or PDT-effective. However, the presence of biomolecules that are part of the cells helps to limit this phenomenon by forming physical conjugates that tend to minimize undesirable aggregation in physiological media. In addition, the improved photobiological performance of zinc complexes contributes to the higher PDI response observed in in vitro studies.

Interest in studying metal phthalocyanines for their photodynamic inhibitory effects against various viruses has been strengthened by the recent global health crisis. The previous study on cationic and anionic Zn(II) phthalocyanines, such as methylpyridiloxy-oxy-substituted Zn(II) phthalocyanine (ZnPcMe), with four substituents in a peripheral position, and tetra-sulfophenoxy-substituted Zn(II) phthalocyanine (ZnPcS), suggested the potential of PDI of DNA-enveloped viruses [25]. These stains were herpes simplex virus type 1 (HSV-1) and vaccinia virus (VV), RNA-enveloped viruses such as bovine viral diarrhea virus (BVDV) and Newcastle disease virus (NDV), and two naked viruses (the enterovirus Coxsackie B1, an RNA-containing virus, and human adenovirus 5, a DNA virus), all affected with a marked, identical virucidal effect against herpes simplex virus type 1 under irradiation for 5 or 20 min (Δlog = 3.0 and 4.0, respectively), independent of charge. The effect was weaker towards the vaccinia virus, wherein Δlog = 1.8 for cationic ZnPc1 and 2.0 for anionic ZnPcS. Bovine viral diarrhea virus exhibited a pronounced sensitivity to ZnPcS at 5 and 20 min of irradiation (Δlog = 5.8 and 5.3, respectively) and only a moderate sensitivity to ZnPc1 (Δlog = 1.8). The complexes did not inactivate Newcastle disease virus, Coxsackievirus B1, and human adenovirus type 5 [32]. Three other cationic phthalocyanine derivatives were tested for antiviral activity with PDI towards the following coated and non-enveloped viruses: bovine viral diarrhea virus (BVDV), influenza virus A (H3N2), poliovirus type 1 (PV-1), and human adenovirus type 5 (HAdV5). Virucidal and irradiation effects were registered toward BVDV by octa-methylpyridiloxy-substituted Ga(III) phthalocyanine (GaPc8), whereas only tetra-methylpyridiloxy-substituted Ga(III) phthalocyanine (GaPcMe) exhibited a remarkable PDI. No effect was observed against the influenza A virus. In contrast, both Ga(III) phthalocyanines exhibited remarkable PDI potential against naked viruses, especially HAdV5, revealing combined virucidal and irradiation effects [33].

In conclusion, TBO directly and to a great extent inactivates bovine coronavirus in vitro after irradiation at 99 J/cm^2^ at 635 nm. Its antiviral activity is pronounced at low concentrations (0.02–0.3 µM), which are not toxic to MDBK cells, leading to complete protection of the cellular monolayer from the viral cytopathic effect. The selectivity index of toluidine blue O (TBO) is very high (170), indicating that it is a selective bioactive compound warranting further pharmacological investigations for its application in veterinary and/or human medical practice.

In our previous study, we found that the phthalocyanine complexes we studied effectively inhibited HSV-1 upon irradiation [18]. In the PDI of the virus that had penetrated the cell, both complexes showed activity, but it was significantly stronger in the case of ZnPcMe with a determined PII = 33.0. Extracellular virions were affected almost equally upon irradiation with both photosensitizers, and the process was time-dependent. At 20 min of irradiation, inhibition with Δlg = 4.5 was recorded for ZnPcMe and Δlg = 4.25 for GaPcMe. In relation to the stage of viral adsorption to the cells, a remarkable effect was shown by GaPcMe with 100% inhibition of viral adsorption upon irradiation throughout the entire period of the process (Δlg = 6.5). In the case of ZnPcMe, the effect was also remarkable, although slightly weaker compared to GaPcMe (Δlg = 4.5). When irradiated at equal but different intervals of viral adsorption, both phthalocyanines showed the same PDI at each stage (Δlg = 3.25). The same result was obtained for ZnPcMe in the present study, for the PDI of HCoV-OC43 virions. When irradiated at equal but different intervals of viral adsorption, the process is affected to the same extent (Δlg = 3.5). This shows that regardless of which stage of viral adsorption the photosensitizer is irradiated and activated at, it causes disturbances in the process to the same extent. A significant difference in our two studies is that for the PDI of extracellular HSV-1, 100% inhibition is observed for GaPcMe, while for HCoV-OC43 the process is affected to a very weak extent (Δlg = 1.0–1.5). With ZnPcMe, HCoV-OC43 adsorption was inhibited 100% by irradiation during the entire process (Δlg = 5.0), while with HSV-1 PDI, little residual attached virus was observed (Δlg = 4.5). Regarding the PDI of extracellular HCoV-OC43, the same time/dose dependence of irradiation was observed as with HSV-1, although the inhibition was generally slightly weaker than with HSV-1. At 20 min of irradiation, a PDI of Δlg = 4.0 was observed with ZnPcMe, and Δlg = 2.5 with GaPcMe. Regarding the PDI of the virus inside the cell, a significantly stronger effect was observed with HCoV-OC43, compared to HSV-1. ZnPcMe demonstrated a PII = 44.0, and GaPcMe a PII = 23.3. This shows that when irradiating the studied photosensitizers in the presence of HCoV-OC43 inside the cell, the process is much more sensitive and more viral structures are damaged compared to HSV-1.

The present study, based on wet-lab findings, suggested that both cationic tetra-substituted ZnPcMe and GaPcMe, bearing four peripheral substituents, possess high antiviral potential. Based on the observed inhibitory activity, the metal complexes were further evaluated for possible interaction with coronavirus-related protein targets. To align the computational analysis more closely with the experimental HCoV-OC43 model, the OC43 3CLpro structure (PDB 9PAM) was included as the primary virus-relevant target. In addition, selected SARS-CoV-2 structural and host targets (Nsp5, Nsp12, M protein, S protein, and ACE2) were examined to explore broader pan-coronavirus interaction patterns and identify possible new mechanistic directions for the phthalocyanines.

Docking analysis suggested that both metal complexes may interact favorably with the selected targets, with ZnPcMe showing slightly lower predicted binding energies than GaPcMe in most cases (Table 5) [34]. Compared with the metal complexes, REM displayed a different interaction pattern, characterized by more conventional hydrogen bonds, whereas the metal complexes relied more heavily on non-conventional contacts [35]. Overall, the interaction profiles were broadly comparable, but these results should be interpreted as tentative hypotheses of target engagement rather than evidence of stronger binding or biological activity. To complement the docking results, molecular dynamics simulations were performed for the selected Nsp12 complex. Nsp12 was chosen for MD because it yielded the most favorable docking scores among the examined targets, making it a suitable representative system for assessing the dynamic stability of the predicted interactions. In contrast, HCoV-OC43 3CLpro (PDB 9PAM) was included primarily to provide virus-specific docking context aligned with the in vitro model.

MD simulations suggested that the complexes did not exhibit major instability under the simulation conditions. RMSD values suggested limited structural deviation over time for all three systems (Figure 14A). RMSF profiles were similar overall, with some localized fluctuations for ZnPcMe and REM that may reflect residue flexibility [36]. Rg values showed no major differences among the complexes, suggesting broadly similar compactness during the simulation (Figure 14C). Hydrogen-bond analysis showed that ZnPcMe typically formed about two hydrogen bonds, GaPcMe alternated between one and two, and REM generally formed three to four. These interaction patterns were generally consistent with the docking observations, although they fluctuated over time (Figure 14D) [26]. Taken together, the MD results provide indicative computational evidence for the persistence of the modeled complexes under the simulation conditions, but they do not establish antiviral efficacy. However, as the simulations were based on single trajectories without independent replicas, these findings should be interpreted as preliminary and not definitive evidence of complex stability. 

The present study has some limitations. So far, the effect has only been observed at the initial stage of viral replication. It can only affect specific viral proteins that have entered the host cell, thereby blocking expression of the viral genome and preventing further stages of viral replication. In addition, the in silico analysis provides only hypothesis-generating insight into possible target engagement. Our subsequent studies will aim to trace the influence of photodynamic inhibition upon irradiation at different stages of the viral replicative cycle, to obtain clearer data on the optimal application of photosensitizers.

## 5. Conclusions

Initially, an antiviral PDI-based method was explored for purifying blood products and for potential nasal disinfection to prevent the spread of coronaviruses. Ga(III)- and Zn(II) phthalocyanine complexes showed a clear inhibition of the viral replication cycle with photoinactivating indices of 44.0 and 23.3, respectively. They also showed remarkable inhibitory activity against HCoV-OC43 virions, as well as their adsorption stage to sensitive Vero E6 cells. This inhibition was particularly pronounced for ZnPcMe, with the observed effect depending on the radiation dose (i.e., the irradiation time). The experimentally obtained data were complemented by molecular modeling. The main virus-relevant target was HCoV-OC43 3CLpro (PDB 9PAM), which was included to align the computational analysis with the in vitro model. In addition, selected SARS-CoV-2 targets (Nsp5, Nsp12, M protein, S protein, and ACE2) were examined as exploratory homologues to broaden the mechanistic perspective. Compared with the co-crystallized ligands where available and REM, the photosensitizing compounds generally showed favorable predicted binding affinities toward several targets. Taken together, these findings suggest that the phthalocyanine complexes may contribute to antiviral activity primarily against HCoV-OC43 and, more tentatively, may also interact with related coronavirus targets; however, these conclusions remain preliminary and require further experimental validation.

## Figures and Tables

**Table 1:** Photodynamic inactivation in dependence on treatment conditions.

Method	Stage of Influence of Viral Particles											
	Extracellular Virions				Virus Adsorption to Cells						Viral Replicative Cycle	
Section 2.9.1	Room temperature			5 min	⇓	37 °C for 60 min				⇑	37 °C for 48 h	
			10 min Room temperature		⇓	37 °C for 60 min				⇑	37 °C for 48 h	
		15 min Room temperature			⇓	37 °C for 60 min				⇑	37 °C for 48 h	
	20 min Room temperature				⇓	37 °C for 60 min				⇑	37 °C for 48 h	
Section 2.9.2.1					⇓	15 min 4 °C				⇑	37 °C for 48 h	
					⇓	30 min 4 °C				⇑	37 °C for 48 h	
					⇓	45 min 4 °C				⇑	37 °C for 48 h	
					⇓	60 min 4 °C				⇑	37 °C for 48 h	
Section 2.9.2.2					⇓	15 min 4 °C				⇑	37 °C for 48 h	
					⇓		15 min 4 °C			⇑	37 °C for 48 h	
					⇓			15 min 4 °C		⇑	37 °C for 48 h	
					⇓				15 min 4 °C	⇑	37 °C for 48 h	
Section 2.9.3					⇓					⇑	20 min 37 °C	37 °C for 120 h

⇓—addition of the viral inoculum, ⇑—removal of unadsorbed virus, 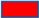
—period of irradiation.

**Table 2:** Cytotoxicity on the Vero E6 cell line and effect on HCoV-OC43 replication of ZnPcMe, GaPcMe and REM.

Photo-Sensitizer	Cytotoxicity					Effect on Viral Replication		
	Dark		Light (LED 660 nm)			Dark	Light (LED 660 nm)	
	CC_50_ _Dark_ Mean ± SD (μg/mL)	MTC _Dark_ (μg/mL)	CC_50 LED 660 nm_ Mean ± SD (μg/mL)	MTC _LED 660 nm_ (μg/mL)	PIF	IC_50 Dark_ (μg/mL)	PIC_50_ _LED 660 nm_ Mean ± SD (μg/mL)	PII
ZnPcMe	24.3 ± 2.8 ***	5.0 ***	2.2 ± 0.5	0.5	11.04	-	0.05 ± 0.003	44.0
GaPcMe	33.6 ± 3.6 ***	5.0 ***	4.1 ± 0.9	0.5	8.2	-	0.8 ± 0.02	23.3
REM	129.2 ± 9.3	50.0	-	-	-	1.4 ± 0.3	-	-

SD—standard deviation; *** *p* ˂ 0.001, comparing the value for each photosensitizer with REM; CC_50_—50% cytotoxic concentration; MTC—maximum tolerable concentration; IC_50_—50% inhibitory concentration; PIC_50_—50% photoinactivating concentration; light-emitting diode (LED); PIF—Photoinhibition Factor; PII—Photoinactivating index; REM—remdesivir.

**Table 3:** Effect of photosensitizers on extracellular HCoV-OC43 virions.

Photosensitizer	Δlg			
	5 min	10 min	15 min	20 min
ZnPcMe	1.75 ± 0.15 ***	2.5 ± 0.2 ***	4.0 ± 0.15 ***	4.0 ± 0.2 ***
GaPcMe	0.75 ± 0.15	1.5 ± 0.15 *	2.0 ± 0.12 ***	2.5 ± 0.3 ***

* *p* ˂ 0.05, comparing the effect of each photosensitizer with the viral control; Student’s *t*-test; *** *p* ˂ 0.001, comparing the effect of each photosensitizer with the viral control; Student’s *t*-test.

**Table 4:** Effect of photosensitizers on the viral adsorption step of HCoV-OC43 to susceptible VeroE6 cells.

Compounds	Δlg			
	First Experimental Setting			
	0–15 min	0–30 min	0–45 min	0–60 min
ZnPcMe	3.5 ± 0.12 ***	3.5 ± 0.2 ***	4.75 ± 0.25 ***	5.0 ± 0.2 ***
GaPcMe	1.0 ± 0.2	1.0 ± 0.15	1.0 ± 0.25	1.5 ± 0.25 *
	Second experimental setting			
	0–15 min	16–30 min	31–45 min	46–60 min
ZnPcMe	3.5 ± 0.2 ***	3.5 ± 0.25 ***	3.5 ± 0.25 ***	3.5 ± 0.33 ***
GaPcMe	1.0 ± 0.2	1.0 ± 0.2	1.0 ± 0.2	1.0 ± 0.2

* *p* ˂ 0.05, comparing the effect of each photosensitizer with the viral control; Student’s *t*-test; *** *p* ˂ 0.001, comparing the effect of each photosensitizer with the viral control; Student’s *t*-test.

**Table 5:** Binding energies, interaction types, and interacting residues of ZnPcMe, GaPcMe, co-crystallized ligands, and remdesivir with the target enzyme structures.

Compound	Target	Binding Energy (kcal/mol)	H-Bonding Residues	Other Interaction Residues
GaPcMe	9PAM	−9.8	-	His41 ^b,e^, Met49 ^b^, Gln164 ^c^, Glu166 ^a(x2)^, Gln189 ^c^, Val191 ^b^
ZnPcMe	9PAM	−9.9	-	His41 ^f^, Met49 ^b^, Cys142 ^b,e^, Cys145 ^c^, Leu165 ^e^, Glu166 ^a(x4)^
ALG-097655	9PAM	−8.4	His141, Cys145, Glu166(x2), Gln192	His41 ^b^, Met49 ^b^, Leu165 ^b^, Glu166 ^a,h^, Leu167 ^h^, Val190 ^h^
ZnPcMe	7QBB	−10.1	Cys85, Arg105	Arg105 ^a(x2)^, Pro184 ^b(x4),c^, Ala193 ^c^
GaPcMe	7QBB	−9.6	Gln189, Ala191	His41 ^b,c^, Cys44 ^b^, Met49 ^b^, Leu141 ^b^, Met165 ^b,d^, Pro168 ^b,e^, Gln189 ^e^
Compound 18	7QBB	−9.2	Asn142, His163, Glu166	Cys44 ^d^, Met49 ^b^, Phe140 ^c^, Leu141 ^g^, Cys145 ^d^
Remdesivir	7QBB	−7.2	Thr24, His41, Asn142(x2)	His41 ^b^, Met49 ^b^, Cys145 ^d^, Met165 ^b^
ZnPcMe	7AAP	−10.6	Leu49, Asn209	Phe35 ^f^, Tyr38 ^c^, His75 ^a^, Val204 ^b^
GaPcMe	7AAP	−11.1	Val204	Phe35 ^e^, Ile37 ^b,e^, Asp40 ^a^, Val204 ^b^, Asp208 ^a^, Asp218 ^a^, Asp221 ^a(x5)^
Remdesivir	7AAP	−7.0	Lys50, Asn52, Asp208, Asp221	Phe35 ^b(x2)^, Ile37 ^b^, Phe48 ^b^, Lys50 ^b^, Asp208 ^a^, Asp218 ^a^, Asp221 ^a,c^
ZnPcMe	7VGR	−10.6	Asn41, Asn117	Lys50 ^b^, Leu54 ^b^, Glu115 ^a^
GaPcMe	7VGR	−10.3	Trp31	Tyr39 ^b^, Lys50 ^b^, Leu54 ^b^, Met91 ^b,d^, Ala98 ^b^, Asn113 ^c^, Pro114 ^b(x2)^, Glu115 ^a^, Thr116 ^c^
Remdesivir	7VGR	−7.3	Met91, Ser94, Ser111, Asn113, Thr116	Leu34 ^b^, Lys50 ^b(x2)^, Trp58 ^f^
ZnPcMe	6VSB	−9.7	Thr167	Ser112 ^c^, Lys113 ^b^, Thr114 ^c^, Val130 ^b^, Glu132 ^a^, Pro230 ^c^, Ile231 ^b^, Ile233 ^b^
GaPcMe	6VSB	−9.1	Thr167	Thr114 ^e^, Glu132 ^a(x3)^, Ile231 ^c^, Ile233 ^b^
Remdesivir	6VSB	−5.8	Pro39, Lys202	Lys41 ^b^, Asp198 ^a^, Tyr200 ^f^
ZnPcMe	1R42	−9.5	His34	Lys26 ^c^, Leu29 ^b^, His34 ^b(x2)^, Glu37 ^a^, Ala387 ^b^, Gln388 ^f^, Pro389 ^b^, Arg393 ^a^
Remdesivir	1R42	−8.6	Gln102, Asp206, Glu208	Gln98 ^c^, Ala99 ^b^, Tyr202 ^b^, Trp203 ^b^

^a^ pi-ion, ^b^ alkyl/pi-alkyl, ^c^ carbon–hydrogen bond, ^d^ pi-sulfur, ^e^ pi-sigma, ^f^ pi-pi, ^g^ amide-pi, ^h^ halogen.

## Data Availability

The raw data supporting the conclusions of this article will be made available by the authors on request.
